# AIDS Vaccine Development: Challenges and Opportunities

**DOI:** 10.3201/eid1311.070790

**Published:** 2007-11

**Authors:** Robert T. Chen

**Affiliations:** * Centers for Disease Control and Prevention, Atlanta, Georgia, USA

**Keywords:** AIDS vaccines, HIV vaccine, clinical trial, book review

A quarter of a century after AIDS became known as a frightening new disease, substantial progress has been attained on treatments that convert this once certain death sentence into a manageable chronic disease. While some prevention successes have been attained (e.g., screening of the blood supply in industrialized countries), a safe and effective vaccine—the “holy grail” of public health prevention—remains elusive.

In the late 1990s, the first HIV vaccine taken to phase III trial, the VaxGen gp120 vaccine (VaxGen, Brisbane, CA, USA), became the basis for substantial debate and controversy between empiricists (generally public health persons who believed that the urgency of the pandemic required taking some risks, including a potentially low-efficacy vaccine as a first step) and reductionists (generally basic scientists and researchers who felt that the gp120 vaccine was unlikely to work given our state of knowledge and who wanted to wait for a better candidate vaccine). With trial results now available, we know that this vaccine was not efficacious. We also know that a phase III trial, although challenging to organize and conduct among persons at high risk, is doable. What else we do and do not know scientifically is summarized nicely in the 19 chapters of this excellently edited, concise (150 pages), softbound book.

The book is organized into 5 parts: Global Overview; What Does a Vaccine Need to Do?; Preclinical Development: Design Challenges; Clinical Trials; and From Testing to Deployment. Each chapter, written by experts in each field, is impressive in its balance of compactness (3–4 double-sided pages, including references), technical content, and user-friendliness (abstract and conclusion for each chapter make quick review easy).

The authors and editors are to be commended for bringing each of the key topics relevant to HIV vaccines to the reader in a highly accessible form. Key topics include HIV pathogenesis; the twists and turns of what specific knowledge of simian immunodeficiency virus and nonhuman primates is or is not applicable to HIV and humans; and the highly technical nature of modern immunology, virology, and structural biology. The editors were careful to include chapters on important nonscientific aspects of HIV vaccine development, such as clinical site development, regulatory issues, scale-up, and manufacturing.

This book provides an excellent introductory overview for the beginning HIV vaccine researcher or any person who needs a more technical primer on the various aspects of the HIV vaccine challenge. The number of HIV vaccine researchers is now increasing, given the support of several organizations (e.g., Bill and Melinda Gates Foundation) and collaborations (e.g., Global HIV Vaccine Enterprise, the Partnership for AIDS Vaccine Evaluation, and the Center for HIV-AIDS Vaccine Immunology). These organizations, collaborations, and researchers are attempting to better organize the human and technical resources needed to challenge this formidable foe on the scale of the Manhattan Project or the March of Dimes search for a polio vaccine. Let us hope that they will eventually succeed.

